# Dynamically Modulating Plasmonic Field by Tuning the Spatial Frequency of Excitation Light

**DOI:** 10.3390/nano10081449

**Published:** 2020-07-24

**Authors:** Sen Wang, Minghua Sun, Shanqin Wang, Maixia Fu, Jingwen He, Xing Li

**Affiliations:** 1 Shandong Provincial Engineering and Technical Center of Light Manipulations & Shandong Provincial Key Laboratory of Optics and Photonic Device, College of Physics and Electronics, Shandong Normal University, Jinan 250014, China; sunqiqihua@163.com (M.S.); wsq8868666@126.com (S.W.); lixing0607@126.com (X.L.); 2 Key Laboratory of Grain Information Processing and Control, College of Information Science and Engineering, Henan University of Technology, Zhengzhou 450001, China; fumaixia@126.com; 3 State Key Laboratory of Space-Ground Integrated Information Technology, Beijing Institute of Satellite Information Engineering, Beijing 100095, China; hejingwen880112@126.com

**Keywords:** surface plasmon polaritons, spatial frequency, Fourier transform, focusing, vortex

## Abstract

Based on the Fourier transform (FT) of surface plasmon polaritons (SPPs), the relation between the displacement of the plasmonic field and the spatial frequency of the excitation light is theoretically established. The SPPs’ field shifts transversally or longitudinally when the spatial frequency components $f_{x}$ or $f_{y}$ are correspondingly changed. The SPPs’ focus and vortex field can be precisely located at the desired position by choosing the appropriate spatial frequency. Simulation results are in good agreement with the theoretical analyses. Dynamically tailoring the plasmonic field based on the spatial frequency modulation can find potential applications in microparticle manipulation and angular multiplexed SPP focusing and propagation.

## 1. Introduction

Resulting from the interaction between light and metal, surface plasmon polaritons (SPPs) are electromagnetic evanescent waves, tightly confined to the interface of dielectric and metal [1,2]. Since the discovery of extraordinary transmission of light through periodic subwavelength metallic structures caused by SPPs [3], SPPs have always been of interest to a wide spectrum of scientists and have become an essential branch of nanophotonics. With the subwavelength and quasi 2D wave features, SPPs offer a potential platform to realize miniaturized photonic circuits [4,5]. SPP devices with various functionalities, including focusing [6,7,8,9], vortex generation [10,11,12,13,14], unidirectional propagation [15,16,17], logical operations [18], nondiffracting beam [19,20,21,22] and hologram [23], have been demonstrated to control the plasmonic field.

The SPPs’ dispersion relation indicates that the momentum of the SPPs is greater than the corresponding free space excitation light [1,2]. To generate SPPs, the prism-coupling technique is firstly developed to bridge the momentum mismatch. However, only at the particular incident angle where the in-plane wavevector component of the excitation light in the prism coincides with the SPPs wavevector at the dielectric/metal interface can SPPs be excited. Another technique for SPP excitation makes use of the diffraction from a topological defect on the metal surface, such as a subwavelength slit or hole. This is based on the fact that diffracted components of light with all wave vectors are present in the near-field region. Under this circumstance, no special incident angle is required to generate SPPs. Most studies adopt the latter technique in the experiments and the excitation light is normally incident on the metal surface for convenience. However, the spatial frequency determined by the incident angle of excitation light can significantly affect the amplitude and phase of the generated SPPs [24,25,26]. By modulating the polarization, amplitude, and phase of the excitation light [15,27,28,29,30,31,32,33], dynamical SPP devices have been extensively studied. The influences of the spatial frequency of incident light on the functionality of SPP devices are less discussed.

In this paper, we first theoretically analyzed the dependence of the displacement of the plasmonic field on the spatial frequency of the excitation light from the view of the SPPs’ Fourier transform (FT). Then, for oblique incident light with different spatial frequencies, the SPPs’ field generated by a plasmonic lens with different structures is studied. The results show that the SPPs’ focus and vortex field will experience a transversal or longitudinal shift when the spatial frequency component $f_{x}$ or $f_{y}$ are changed. The SPPs’ field can be precisely generated at the desired position by choosing the appropriate spatial frequency. The consistency between the simulation results and theoretical analyses verifies the feasibility of the method. The proposed versatile approach may inspire the realization of other dynamic SPP devices based on spatial frequency modulation and suggest charming applications, including microparticle manipulation and angular multiplexed SPP focusing and propagation.

## 2. Results and Discussions

**Figure 1:**
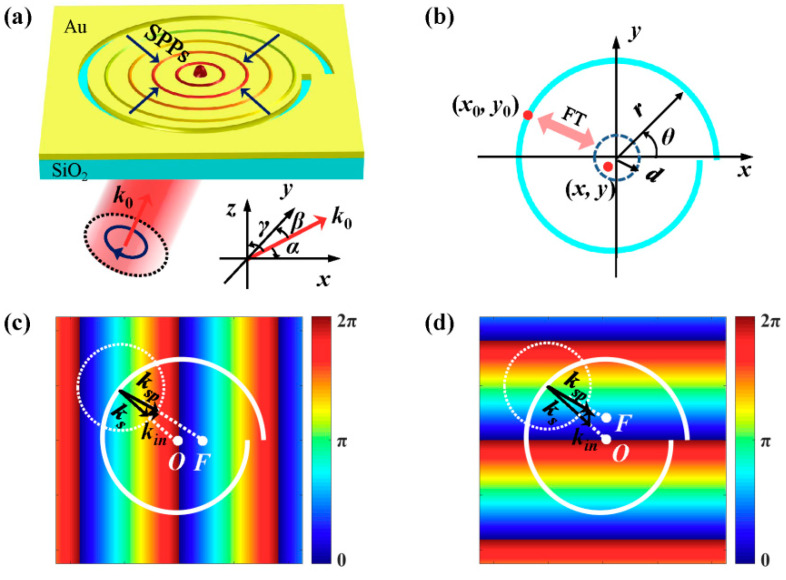
(**a**) Schematic diagram of a plasmonic lens illuminated by obliquely incident light. (**b**) presents the FT relation between the SPPs’ field along the Archimedes spiral slit and the SPPs’ field near the origin. (**c**,**d**) schematically show the linear gradient phase imprinted on the excited SPPs and the displacements of SPPs’ focus.

As shown in Figure 1a, the structure of the plasmonic lens is an Archimedes spiral slit etched on a 200-nm-thick Au film, and the substrate is SiO_2_. Right circularly polarized light is incident on the plasmonic lens obliquely and the excited surface plasmon polaritons (SPPs) are focused around the origin. The angles between the wave vector $k_{0}$ and the *x*-axis, *y*-axis, and *z*-axis are denoted as *α*, *β*, and *γ*, respectively. Thus, the spatial frequency of the excitation light can be written as:

(1) $$f_{x}=\cos\alpha /\lambda_{0},\;f_{y}=\cos\beta /\lambda_{0},\;\mathrm{and}\;f_{z}=\cos\gamma /\lambda_{0}$$

The geometry of the Archimedes spiral slit is mathematically defined as $r=r_{0}-\left(\theta \lambda_{sp}\right)/2\pi$, where r is the radius of the spiral slit, $r_{0}$ is the initial radius, 0 ≤ θ ≤ 2π is the azimuthal angle, and $\lambda_{sp}$ is the wavelength of SPPs, as illustrated in Figure 1b. Based on the Huygens Fresnel principle for SPPs [34], the SPPs’ distribution in the vicinity of the origin *O* (d ≪ r) can be regarded as the Fourier transform (FT) of the complex amplitude $E_{sp}\left(x_{0},y_{0}\right)$ along the spiral slit [35,36]. The FT relationship is given by:

(2) $$E_{sp}\left(x,y\right)=ℱ\left\{E_{sp}\left(\frac{x_{0}}{r\lambda_{sp}},\frac{y_{0}}{r\lambda_{sp}}\right)\right\}.$$

The obliquely incident plane wave is expressed as $E\left(x,y\right)=\exp[i2\pi \left(f_{x}x+f_{y}y+f_{z}z\right)]$ and the phase distribution on the *xy*-plane (*z* = 0) is $\Phi \left(x,y\right)=2\pi \left(f_{x}x+f_{y}y\right)$. Considering that the phase of the incident light can be transferred directly to the excited SPPs [29,31], the phase of the excited SPPs along the spiral slit should be:

(3) $$\Phi_{sp}\left(x_{0},y_{0}\right)=2\pi \left(f_{x}x_{0}+f_{y}y_{0}\right)=\frac{2\pi }{\lambda_{0}}\left(\cos\alpha x_{0}+\cos\beta y_{0}\right)$$

which reveals that the spatial frequency of the incident light can affect the phase of excited SPPs. When the excitation light illuminates the slit obliquely, the SPPs will be imprinted with an additional linear gradient phase, and the focus will deviate from the center. Substituting the Equation (3) into Equation (2), we obtain:

(4) $$\begin{array}{l} ℱ\left\{E_{sp}\left(\frac{x_{0}}{r\lambda_{sp}},\frac{y_{0}}{r\lambda_{sp}}\right)\exp\left[i\frac{2\pi }{\lambda_{0}}\left(\cos\alpha x_{0}+\cos\beta y_{0}\right)\right]\right\}\\ =\iint E_{sp}\left(\frac{x_{0}}{r\lambda_{sp}},\frac{y_{0}}{r\lambda_{sp}}\right)\exp\left[i\frac{2\pi }{\lambda_{0}}\left(\cos\alpha x_{0}+\cos\beta y_{0}\right)\right]\exp\left[-i\frac{2\pi }{r\lambda_{sp}}\left(xx_{0}+yy_{0}\right)\right]dx_{0}dy_{0}\\ =\iint E_{sp}\left(\frac{x_{0}}{r\lambda_{sp}},\frac{y_{0}}{r\lambda_{sp}}\right)\exp\left\{-i\frac{2\pi }{r\lambda_{sp}}\left[\left(x-\frac{\lambda_{sp}r\cos\alpha }{\lambda_{0}}\right)x_{0}+\left(y-\frac{\lambda_{sp}r\cos\beta }{\lambda_{0}}\right)y_{0}\right]\right\}dx_{0}dy_{0}\\ =E_{sp}\left(x-\frac{\lambda_{sp}r\cos\alpha }{\lambda_{0}},y-\frac{\lambda_{sp}r\cos\beta }{\lambda_{0}}\right)\\ =E_{sp}\left(x-\lambda_{sp}rf_{x},y-\lambda_{sp}rf_{y}\right) \end{array}$$

From Equation (4), we can conclude that the SPPs’ focus generated by obliquely excitation light will experience a longitudinal displacement δy and a transversal displacement δx:

(5) $$\delta x=\lambda_{sp}rf_{x},\;\delta y=\lambda_{sp}rf_{y}$$

In [24,37], the SPPs’ focus excited by the obliquely incident light are studied for the circular plasmonic lens. Different from the analyses based on the momentum conservation [24] or optical path theory [37], we adopt the FT of SPPs, and the obtained Equation (5) clearly reveals the relation between the spatial frequency of incident light and the shift of the SPPs’ field. The result can be utilized to analyze not only the SPPs’ focusing field generated by the spiral slit and semicircular slit but also the SPPs’ vortex field generated by the circular slit.

When the wave vector of incident light is in parallel with the *xz*-plane (β=90°), the excited SPPs are imposed with a transversal gradient phase in Figure 1c, and the SPPs’ focus *F* shifts along the *x*-axis direction. Similarly, the SPPs’ focus shifts longitudinally for excitation light propagating in the *yz*-plane (α=90°). Figure 1d schematically presents the longitudinal gradient phase of the corresponding incident light. In the momentum space, the displacement of the SPPs’ focus can also be qualitatively explained from the point view of in-plane wave vector matching [38]. The magnitude of SPPs’ wave vector $k_{sp}$ is a constant determined by the dispersion curve. The in-plane wave vector component of incident light $k_{in}=k_{0}\sin\gamma$ can influence the direction of $k_{sp}$. Besides, due to the scattering of the incident light, the spiral slit can provide a wave vector $k_{s}$, which points to the origin *O* [24]. To excite SPPs, the three wave vectors should satisfy $k_{sp}=k_{in}+k_{s}$, as presented in Figure 1c,d. For normally incident light, the in-plane wave vector component is zero, and the SPPs’ wave vector $k_{sp}=k_{s}$ points to the center. Therefore, the excited SPPs are focused in the origin *O*. When the wave vector of incident light is not perpendicular to the metal surface, the in-plane wave vector $k_{in}$ along the *y*-axis or *x*-axis will make $k_{sp}$ deviate from the radial direction. And the SPPs’ focus will shift transversely or longitudinally.

**Figure 2:**
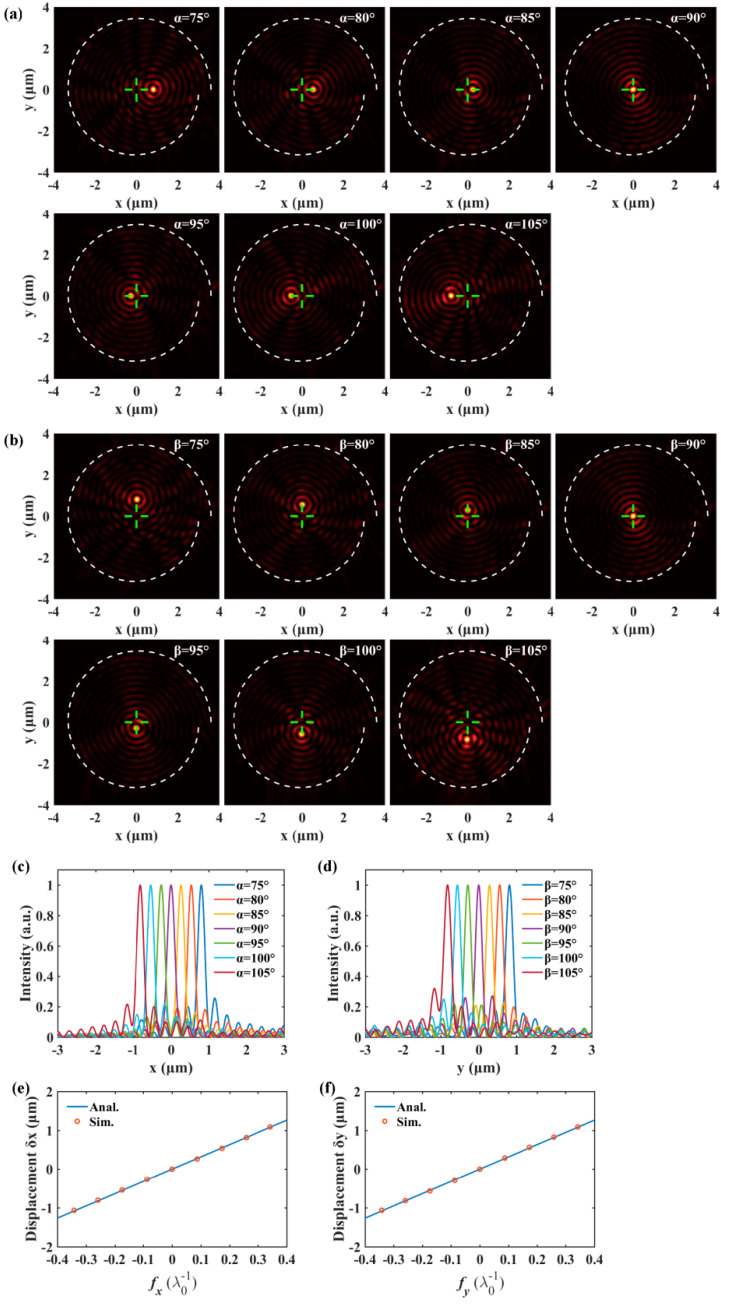
(**a**,**b**) show that the SPPs’ focus will shift transversally or longitudinally when the spatial frequency component $f_{x}$ or $f_{y}$ changes. (**c**,**d**) are the profiles of the SPPs’ focus generated by light with different incident angles. (**e**,**f**) compare the theoretical displacements of SPPs’ focus with the simulated values.

Numerical simulations based on the finite difference time domain method (Lumerical FDTD Solutions) are performed to study the focusing properties of SPPs excited by oblique incidence. In the simulations, the spiral slit can be obtained from the build-in object library, and the initial radius and the width of the slit are set as 3.5 μm and 200 nm, respectively. The mesh accuracy 3 is utilized and the corresponding size of each mesh cell is about 13 × 13 × 40 nm, which can achieve a good tradeoff between accuracy, memory requirements, and simulation time. Perfect matched layers are added in the *x*, *y*, and *z* directions to absorb the propagating SPPs’ fields. With the script function, we defined user custom plane wave, which illuminates the slit from the substrate side. To obtain the distribution of the SPPs’ field, a field monitor is placed 50 nm above the gold film, which is within the decay length of SPPs. The wavelength of the incident light is $\lambda_{0}$ = 632.8 nm and the corresponding wavelength of the SPPs is $\lambda_{sp}$ = 606 nm, calculated by the dispersion relation of SPPs. Considering that an arbitrary wavevector in the *xy* plane can be decomposed into the $f_{x}$ and $f_{y}$ components, we firstly discuss the two basic cases shown in Figure 1c,d ($f_{x}=0$ or $f_{y}=0$). According to Equation (5), when the wave vector of excitation light lies in the *xz*-plane, the spatial frequency component $f_{y}$ is zero (β=90°) and the position of the SPPs’ focus is solely determined by the $f_{x}$ component. Simulated SPPs’ intensity distributions in Figure 2a show that the focus of SPPs is located exactly at the center for normally incident light (α=90°), because the dynamic spiral phase induced by the different optical path and the geometric spiral phase resulting from the interaction between excitation light and slit cancel each other out [12]. However, for obliquely incident light, the additional linear phase presented by Equation (3) makes the focus deviate from the center. Concretely, the SPPs’ focus shifts rightward with the decrease of the angle *α* and the SPPs’ focus shifts leftward with the increase of the angle *α*. Green cross lines are drawn through the center to show the displacements of the focuses clearly. The spiral white dot lines represent the spiral slits. Another case is that the wave vector of incident light is in parallel with the *yz*-plane, which means α=90° and $f_{x}$ = 0. Therefore, the deviations of the SPPs’ focus should be along the *y*-axis based on Equation (5). It can be seen from the simulation results in Figure 2b that the SPPs’ focus shifts downward when the angle *β* rises from 90° to 105° and the SPPs’ focus shifts upward when the angle *β* decrease from 90° to 75°. The profiles of the SPPs’ focus along cut lines x = 0 and y = 0 are extracted and presented in Figure 2c,d to show the positions of the focuses more clearly. Displacements of the SPPs’ focus δx (δy) change from −1.25 μm to 1.25 μm as the angle *α*(*β*) increases from 75° to 105°. The full widths at half maximum (FWHM) of the SPPs’ focus remain about the same (~220 nm) for different incident angles. The displacements of the focuses versus the spatial frequency of excitation light are plotted in Figure 2e,f in which a linear relationship between them can be observed. Theoretical displacements obtained by Equation (5) are in good agreement with the simulated values. Since radius of the spiral slit is not a constant value, a mean value $\bar{r}=\left[r(0)+r(2\pi )\right]/2$ is adopted in the theoretical calculation. The simulation results in Figure 2 are in analogy with the shift theorem of FT in the 3D free space [39]. A linear phase in the frequency domain can lead to the shift in the space domain. Classical optical theories have been extensively exploited to modulate the SPPs’ fields ranging from focusing [6,7,8,9], vortex [10,11,12,13,14] to nondiffracting beam [19,20,21,22], and hologram [23]. However, in the visible frequency range, the wavelength of excitation light differs from the wavelength of excited SPPs. Equation (5) indicates that the linear-gradient phase carried by incident light leads to the shift of SPPs field, in which case two kinds of optical waves are involved.

**Figure 3:**
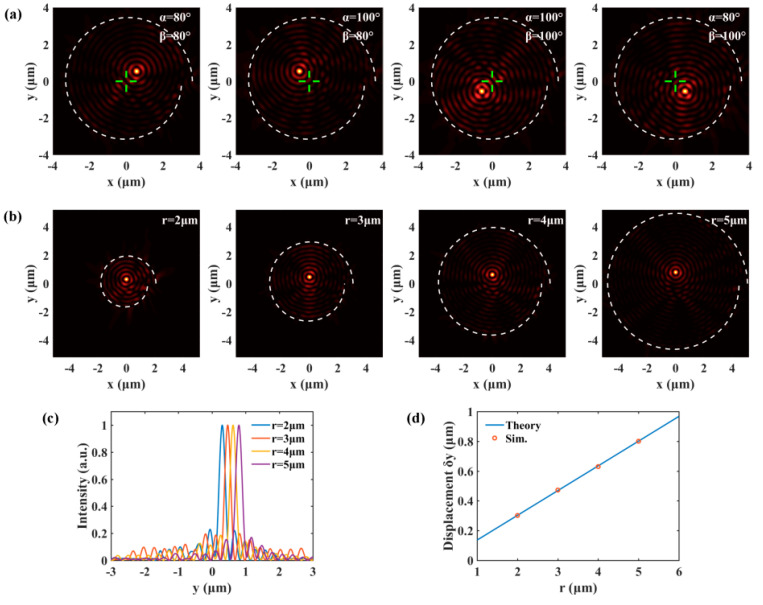
(**a**) shows that the SPPs’ focus can be selectively generated in any quadrant by setting the spatial frequency of excitation light. (**b**) are SPP fields generated by spiral slits with different initial radiuses. (**c**) presents the longitudinal profiles of SPP focuses. In (**d**), we plot the displacement of SPPs’ focus versus radius.

For the general case, the wave vector of incident light lies in neither the *xz*-plane nor the *yz*-plane. The spatial frequency components $f_{x}$ and $f_{y}$ are nonzero, thus the SPPs’ focus should shift both transversally and longitudinally. In Figure 3a, we show that the position of SPPs’ focus can be selectively located in one of the four quadrants. For instance, when $f_{x}$ and $f_{y}$ are positive, the displacements δx and δy are positive, and thus the SPPs’ focus will be located in the first quadrant. The radius of the spiral slit can also affect the displacement of SPPs’ focus according to Equation (5). The distributions of SPPs’ field generated by spiral slits with different initial radiuses are presented in Figure 3b and the incident angle is fixed at α= 90° and β=80°. The longitudinal profiles of SPP focuses in Figure 3c more clearly show that the displacements enlarge with the increase of the radius. It can be seen from the comparisons in Figure 3d that the simulated values are consistent with the theoretical results.

**Figure 4:**
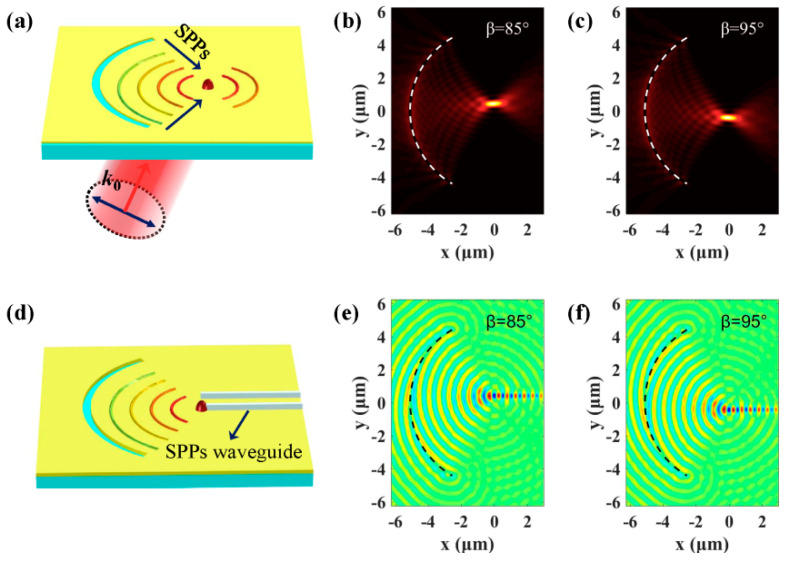
(**a**) Schematic diagram of an arc-shaped slit plasmonic lens illuminated by oblique incident light. (**b**,**c**) are the intensity distributions of SPPs generated by the β= 85° and β= 95° excitation light. (**d**) Two SPP waveguides are added to realize angular multiplexed SPP propagation. The real parts of SPPs in (**e**,**f**) show that the SPPs can be selectively coupled to the upper or lower SPP waveguide by changing the spatial frequency.

The SPPs’ focus generated by a plasmonic lens with different structures can also be dynamically modulated by tuning the spatial frequency of the incident light. Figure 4a schematically presents an arc-shaped slit, which is a kind of commonly used structure to focus SPPs [7,36,40]. The FT relationship given by Equation (2) still holds and, therefore, Equation (5) can be utilized to estimate the displacement of the SPPs’ focus generated by linearly polarized oblique incident light. Simulated intensity distributions in Figure 4b,c show that the SPPs’ focus will experience longitudinal shifts when the spatial frequency $f_{y}$ (α=90°) is changed. For the *r* = 5 μm arc-shaped slit illuminated by the β= 85° and β= 95° incident light, the distance of the displacements is |δy| = 0.426 μm, which agrees well with the theoretical value |δy| = 0.417 μm. This feature of the arc-shaped plasmonic lens can be taken advantage of to realize angular multiplexed SPP propagation. As schematically shown in Figure 4d, two SPP waveguides made up of PMMA [41] are separately added at the two SPPs’ focus generated by the β= 85° and β= 95° excitation light. The real parts of SPPs are given in Figure 4e,f, which show that the SPPs can be selectively coupled to the upper or lower SPP waveguide by changing the incident angle.

**Figure 5:**
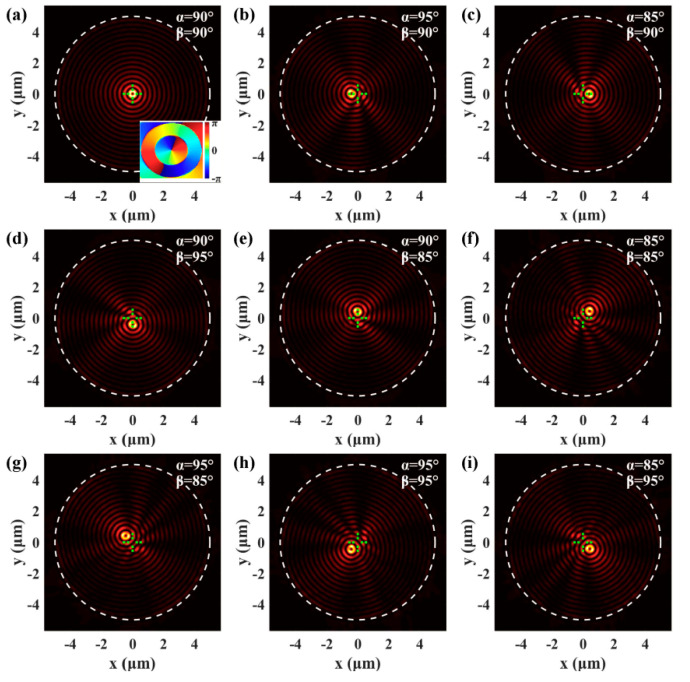
(**a**) For a circular plasmonic lens illuminated by normally incident light, the SPPs’ vortex is excited in the center. (**b**–**e**) show that the SPPs’ vortex moves along the *x*-axis or the *y*-axis when the spatial frequency $f_{x}$ or $f_{y}$ is changed. The SPPs’ vortex can be selectively generated in the first (**f**), second (**g**), third (**h**), or fourth (**i**) quadrant by modulating the spatial frequency.

Furthermore, besides the SPPs focusing field, the SPPs’ vortex can be dynamically controlled by changing the spatial frequency of the excitation light as well. For the circular slit plasmonic lens indicated by the white dot line in Figure 5a, the SPPs’ vortex with a topological charge of *l* = 1 is formed in the center because of the spin–angular momentum carried by the normally incident circularly polarized light. The inset of Figure 5a presents the spiral phase of the SPPs’ vortex and the radius of the circular slit is *r* = 5 μm. When the spatial frequency $f_{x}$ is changed, the SPPs vortex moves along the *x*-axis, which can be observed from Figure 5b,c. The distance of the displacements are |δx| = 0.413 μm for the α= 85° and α= 95° incident light, which is in good agreement with the theoretical value |δx| = 0.417 μm. Figure 5d,e show that the SPPs’ vortex will correspondingly move along the *y*-axis when the spatial frequency $f_{y}$ is changed. By simultaneously modulating the $f_{x}$ and $f_{y}$ components, the SPPs’ vortex can be selectively generated in the first, second, third, or fourth quadrant, as presented in Figure 5f–i. SPPs with field-confinement and enhancement features are effective tools to realize microparticle manipulation. The plasmonic focusing field can trap the microparticle in the center [12] and the plasmonic vortex field can rotate the microparticle around the center [42]. The position of the microparticle is determined by the position of the SPPs’ focus or vortex. The displacement of the SPPs’ focus or vortex means that the microparticle will move as well. Therefore, the microparticle can be selectively moved to the desired position by setting spatial frequency of the incident light according to Equation (5).

## 3. Conclusions

In conclusion, we theoretically revealed the influence of the spatial frequency of excitation light on the plasmonic field based on the FT of SPPs. With oblique incident light, the additional linear-gradient phase results in the deviation of the plasmonic field from the center. For a plasmonic lens with different structures, including the spiral slit, arc-shaped slit, and circular slit, the simulation results all show that the SPPs’ focus or vortex field shifts transversally or longitudinally by tuning the spatial frequency component $f_{x}$ or $f_{y}$, which is consistent with theoretical analyses. Recently, polarization-based dynamical SPP modulation [12,22,27,30,40] and wavelength-multiplexed SPP devices [6,9] have been extensively studied. These techniques usually take advantage of two orthogonal polarization states (left circularly polarized light and right circularly polarized light) or several different wavelengths of excitation light. Thus, the SPPs’ field can only be modulated discontinuously. With the proposed approach, the position of plasmonic field can be continuously modulated because the spatial frequency of excitation light can be continuously tuned. Therefore, spatial frequency-based dynamical SPP modulation is more versatile and multifunctional. Moreover, replacing the metal film with 2D materials, such as graphene or MoS_2_ whose optical properties can be electrically tuned, has effectively facilitated the dynamical modulation of SPPs [43,44]. The strong coupling between plasmon and material excitons [45] has been applied to plasmon-enhanced spectroscopy. Combining the 2D materials and the proposed approach, a more flexible control over SPPs’ fields can be achieved in the future.
